# NKp44 and Natural Cytotoxicity Receptors as Damage-Associated Molecular Pattern Recognition Receptors

**DOI:** 10.3389/fimmu.2015.00031

**Published:** 2015-02-02

**Authors:** Nathan C. Horton, Porunelloor A. Mathew

**Affiliations:** ^1^Department of Cell Biology and Immunology, Institute for Cancer Research, University of North Texas Health Science Center, Fort Worth, TX, USA

**Keywords:** NK cells, natural cytotoxicity receptors, NKp44, DAMPs, tumor ligands

## Abstract

Natural killer (NK) cells are a key constituent of the innate immune system, protecting against bacteria, virally infected cells, and cancer. Recognition and protective function against such cells are dictated by activating and inhibitory receptors on the surface of the NK cell, which bind to specific ligands on the surface of target cells. Among the activating receptors is a small class of specialized receptors termed the natural cytotoxicity receptors (NCRs) comprised of NKp30, NKp46, and NKp44. The NCRs are key receptors in the recognition and termination of virally infected and tumor cells. Since their discovery over 10 years ago, ligands corresponding to the NCRs have largely remained elusive. Recent identification of the cellular ligands for NKp44 and NKp30 as exosomal proliferating cell nuclear antigen (PCNA) and HLA-B-associated transcript 3 (BAT3), respectively, implicate that NCRs may function as receptors for damage-associated molecular pattern (DAMP) molecules. In this review, we focus on NKp44, which surprisingly recognizes two distinct ligands resulting in either activation or inhibition of NK cell effector responses in response to tumor cells. The inhibitory function of NKp44 requires further study as it may play a pivotal role in placentation in addition to being exploited by tumors as a mechanism to escape NK cell killing. Finally, we suggest that the NCRs are a class of pattern recognition receptors, which recognize signals of genomic instability and cellular stress via interaction with the c-terminus of DAMP molecules localized to the surface of target cells by various co-ligands.

## Introduction

Natural killer (NK) cells are a fundamental component of the innate immune system, capable of recognizing and destroying tumor cells as well as cells infected by viruses or bacteria (1, 2). NK cells also secrete cytokines such as interferon-γ (IFN-γ) and thus regulate the function of other immune cells. Furthermore, NK cells play an important role in adaptive immunity by modulating dendritic cell function and recent findings demonstrate that NK cells have memory (3, 4). The ability of NK cells to kill target cells and secrete cytokines is regulated by a delicate balance of activating and inhibitory signals received through distinct classes of receptors found on their cell surface. The balance of signals delivered by those receptors governs NK cell activation, proliferation, and effector functions (5–8). Traditionally, inhibitory killer cell immunoglobulin like receptors (KIRs) and killer cell lectin-like receptors (KLRs) bind cell surface human leukocyte antigen class I (HLA I) molecules expressed by healthy human cells and signal through immunoreceptor tyrosine-based inhibitory motifs (ITIMS) (9–11). When HLA I interacts with inhibitory receptors, dominant inhibitory signaling transmitted by ITIMS prevents activation and cytotoxic action by the NK cell against normal, healthy cells of the body. NK cells may also be inhibited by cytokines released by regulatory cells of the immune system, such as regulatory T cells and myeloid suppressor cells (12).

Activating receptors, including the natural cytotoxicity receptors (NCRs), NKG2D, and 2B4, bind ligands induced by cellular stress, infection, or tumor transformation (13–16). Activating signals are transmitted through immunoreceptor tyrosine-based activating motifs (ITAMs) located in the cytoplasmic tail of the receptor or through ITAMs in adaptor molecules, which associate with activating receptors at the cell surface (8, 17). Therefore, when a target cell lacks or under expresses HLA I and/or over expresses activating ligands, NK cells eliminate that target by releasing preformed cytotoxic granzymes and perforin stored as granules or activate apoptosis pathways in the target cell (8, 18).

## Natural Cytotoxicity Receptors

Among the activating receptors is a specialized group of receptors called the NCRs, which play a key role in recognition and killing of tumor and virally infected cells. Comprising the NCRs are the NKp44, NKp30, and NKp46 receptors. Binding of one or more of these receptors with a specific ligand induces strong NK cell activation and cytotoxicity (19). For optimal recognition and elimination of target cells, the NCRs work best as a team when identifying potential targets (20). This is evident through increased cytotoxicity when multiple NCRs are triggered versus an individual receptor, suggesting simultaneous NCR ligand expression on target cells (20, 21). Several studies have identified and characterized NCR ligands. NKp46 recognition of a ligand on tumor cells has been shown to play a role in prevention of tumor metastasis (22, 23). NKp30 is known to bind B7-H6, a member of the B7 family expressed exclusively on tumor cells (24). While many NCR ligands remain unidentified, they are believed not to be expressed by normal cells but induced by cellular stress or pathological conditions (14).

## NKp44

NKp44 is unique and significant for several reasons. First, expression of the receptor is restricted to activated NK cells capable of initiating an immediate cytotoxic response (25). Second, NKp44 activating function is implicated in HIV-related T cell decline as expression of an activating ligand for NKp44 is induced in uninfected CD4 T cells by the gp41 envelope protein of HIV (26). Earlier studies have shown recognition of viral hemagglutinins of influenza virus by NKp44 enhanced killing of infected cells (27). Finally, NKp44 expression is responsible for a dramatic increase in killing of many tumor cell lines and cross linking the receptor results in the release of cytotoxic granules, IFN-γ, and TNF-α (25, 28–30). While only found on activated NK cells in circulation, NKp44 is constitutively expressed by a specialized subset of NK cells in the decidua, implicating a role for NKp44 during placentation (25, 31, 32). NKp44 is also expressed on a subset of interferon-producing cells located in human tonsils and ILC3 cells in mucosal-associated lymphoid tissues and human decidua (33–37). Recently, it has been shown that NKp44 is indeed functional in ILC3 and its engagement results in TNF but not in IL-22 production (38).

Crystallographic structure of NKp44 demonstrates a surface groove made by two facing β hairpin loops extending from the Ig fold core stabilized by a disulfide bridge between Cystine 37 and Cystine 45 (39). The Ig domain contains an arrangement of positively charged residues at the groove surface, suggesting that NKp44 ligands are anionic (39). Also, the groove appears wide enough to host a sialic acid or an elongated branched ligand. Interestingly, the cytoplasmic tail of NKp44 contains a tyrosine sequence resembling an ITIM (25, 40). Contrary to initial reports, this motif is functional and inhibits the release of cytotoxic agents and IFN-γ (25, 30, 40). NKp44 surface expression is dependent on its association with the ITAM containing DAP 12 accessory protein linked to NKp44 through Lysine 183 in the transmembrane domain (25). Upon recognition of activating ligands, signaling transduced through the ITAMs in Dap 12 result in release of cytotoxic agents, tumor necrosis factor-α, and IFN-γ (29, 40).

While NK cells utilize NKp44 to recognize and kill targets, tumors may also exploit NKp44 to escape NK cell recognition. By engaging NKp44, as well as the other NCRs, tumors can induce NK cell death via up regulation of Fas Ligand in the NK cell, inducing Fas-mediated apoptosis (41). Tumors may also downregulate NKp44 surface expression by shedding soluble MHC Class I chain-related molecules or by releasing indoleamine 2,3-dioxygenase and prostaglandin E2 (42, 43). The latter two molecules are released by mesenchymal stem cells as well, inhibiting NKp44 expression in the tumor microenvironment (44). Additionally, tumors can regulate NKp44 ligand expression to escape NK cell killing, as is the case with acute myeloid leukemia (45). Finally, tumor cells may induce expression of exosomal proliferating cell nuclear antigen (PCNA) when physically contacted by NKp44 expressing NK cells to inhibit NK cell effector function (30).

In addition to its role in immunity, NKp44 also has roles during pregnancy. Decidual NK cells (dNK) make up 50–90% of lymphocytes in the uterine mucosa during pregnancy and constitutively express NKp44 (36, 46, 47). Trophoblast cells and maternal stromal cells of the decidua both express unidentified NKp44 ligands (46). This ligand may be PCNA as the protein is over expressed in trophoblast cells during the first trimester (48). As an inhibitory ligand for NKp44, extracellular PCNA expression on trophoblast cells would help explain the diminished ability of dNK cells to lyse trophoblasts despite low levels of classical HLA I expression (47).

## NKp44 Tumor Ligands

NKp44 is implicated in recognition and killing of numerous types of cancer: neuralblastoma, choriocarcinoma, pancreatic, breast, lung adenocarcionma, colon, cervix, hepatocellular carcinoma, Burkitt lymphoma, diffuse B cell lymphoma, prostate (15, 21, 28). While most of these ligands have not been identified, they appear to be cell cycle regulated, with down regulation of expression during mitosis (28). Recognition of tumor cells is partially mediated through charged-based binding of NKp44 with heparan sulfate proteoglycans (HSPGs) on the surface of tumor cells (49–51). Of note, recognition of HSPG only evokes IFN-γ release by NK cells, not cellular cytotoxicity (49). Thus, HSPGs are believed to only be a co-ligand for NKp44 as well as the other NCRs, potentially facilitating binding with other cellular ligands.

Proliferating cell nuclear antigen is the inhibitory tumor ligand for NKp44 (15, 30). PCNA is a nuclear protein found in all replicating cells, which encircles DNA and increases processivity of DNA replication, but is also involved in DNA repair and cell cycle control (52). NKp44 recognizes PCNA expressed on exosomes shuttled to the surface of tumors cells when in contact with NK cells (15, 30). Recognition of cell surface PCNA colocalizing with HLA I on the cell surface inhibits NK cell cytotoxicity and IFN-γ release (15).

A truncated isoform of mixed-lineage leukemia-5 (MLL5) is an activating cellular ligand for NKp44 (53). This MLL5 isoform contains a specific exon encoding a C-terminus, which interacts with NKp44 (53). Typically located only in the nucleus, MLL5 is a lysine methyltransferase implicated in hematopoietic differentiation and control of the cell cycle (53). Contrary to normal MLL5, the isoform recognized by NKp44 is not found in the nucleus but in the cytoplasm and endoplasmic reticulum, destined to be expressed at the cell surface (53). While MLL5 is expressed in normal tissue, the isoform recognized by NKp44 is only present on tumor and transformed cells (53).

## NCR Co-Ligands

Heparan sulfate proteoglycans have been identified as co-ligands involved in the recognition of tumor cells by the NCRs (49, 50, 54). HSPGs are complex glycoproteins found at the cell surface of mammalian cells or in the extracellular matrix (55, 56). Heparan sulfate is characterized by chains of disaccharide units of *N-*acetyl-d-glucosamine linked to d-glucuronic acid (55, 57). Interestingly, each NCR recognizes distinct forms of heparan sulfate epitopes on HSPGs, specifically highly sulfated microdomains on disaccharide units (58). 2-O-sulfation of iduronic acid and *N*-acetylation of glucosamine on HSPGs are important for interaction with NKp44 (50). NKp30 and NKp46 recognize HSPGs with 2-O-sulfation of iduronic acid and either 6-O-sulfation or 6-N-sulfation of glucosamine (50). Interactions between the NCRs and HSPGs are charge based as each NCR contains basic amino acid residues in their binding cleft and HSPGs are heavily charged molecules.

In addition to HSPGs, HLA I may also serve as a co-ligand for the NCRs. We have shown that HLA I and the NKp44 inhibitory ligand, PCNA, associate on the cell surface (15). In our search to identify a ligand for NKp44, several key pieces of evidence suggested that HLA I plays a role in ligand formation. First, HLA I has been demonstrated to coimmunoprecipitate with anti-NKp44 antibodies; reciprocally, NKp44 coimmunoprecipitates with anti-β-2-microglobulin antibodies (59). Additionally, the Nef protein of HIV prevents surface expression of NKp44 ligand isoform of MLL5 on CD4-infected T cells, which is also consistent with the ability of Nef to retain HLA I intracellularly (60, 61). Finally, the NKp30 ligand, Bat3, colocalizes with HLA I on the extracellular membrane of tumor cells, activating NK cell effector functions (62, 63). Interestingly, all 50 alleles of HLA Class A, B, and C molecules harbor an Asparagine at position 86, close to the residues on the α1 helix, which determine interactions with human NK receptors (64). This site allows for attachment of N-linked glycan structures, which could enable binding of other proteins (64). Electron density mapping of the HLA I glycan structure suggests that it is flexible and could serve as a ligand for other receptors or block access to HLA I molecules. Additionally, HLA-A and -B are almost exclusively disialylated, resulting in these molecules having a negative charge, a characteristic of NKp44 ligands (64). This negative charge combined with a protruding oligosaccharide could potentially facilitate interactions with NKp44.

As co-ligands, HSPGs and HLA I most likely facilitate binding of other proteins, which together form a complex ligand recognized by the NCRs. The most prevalent interaction between HSPGs and other proteins is charged based via clusters of positively charged amino acids on proteins forming ionic bonds with negatively charged sulfate and carboxyl groups on HSPGs (57). HSPGs may offer two mechanisms facilitating NCR ligand recognition. First, HSPGs may bind soluble proteins, which as a whole serve as ligands for the NCRs. Second, HSPGs could bind a soluble protein and an NCR separately, and then act as scaffolding to bring the NCRs into contact with a soluble protein. In the same manner, the protruding oligosaccharide of HLA I, or other regions, may enable assimilation of small proteins or DAMPs.

## DAMPs and the C Terminus

Immune responses are initiated by pattern recognition receptors, which recognize microbial-derived products called pathogen-associated molecular pattern molecules (65). In a similar manner, pattern recognition receptors also recognize molecules released by dying or damaged cells, termed damage-associated molecular pattern (DAMP) molecules or alarmins (66, 67). Recognition of DAMPs contributes to the induction of inflammation, even in the absence of pathogens (68). Normally residing in the nucleus, cytoplasm, or exosomes, DAMPs lack secretion signals but can be actively secreted by non-classical pathways or passively released by necrotic cells (67). DAMPs thus serve as endogenous danger signals when improperly released from damaged cells as well as tumors and activate innate immune cells (67). DAMPs are most often released after trauma, ischemia, or other tissue damage and initiate early inflammatory responses (67). By recruiting immune cells and promoting the release of proinflammatory mediators, DAMPs activate immune responses and initiate pathways leading to tissue repair and regeneration (67, 69). Heat shock proteins, high-mobility group box 1 (HMGB1), S100 proteins, hyaluronan, and heparan sulfate represent a few DAMPs known to date (68).

Like PAMPs, DAMPs are also recognized by pattern recognition receptors. In addition to binding PAMPs, the Toll-like receptors (TLRs) also recognize HMGB1 and a member of the S100 family (67). These two DAMPs are also recognized by the receptor for advanced glycation end products (RAGE) (67). Like the TLRs, RAGE is expressed on numerous immune cells and induces NF-κB-mediated production of cytokines (67). Interestingly, DAMP molecules such as high-mobility group protein B1 and S100A8/9 have the ability to bind heparin sulfate and HSPGs, which are known to be co-ligands involved in NCR-dependent recognition of tumor cells resulting in secretion of IFN-γ but not cytotoxicity (49, 50, 67).

**Figure 1:**
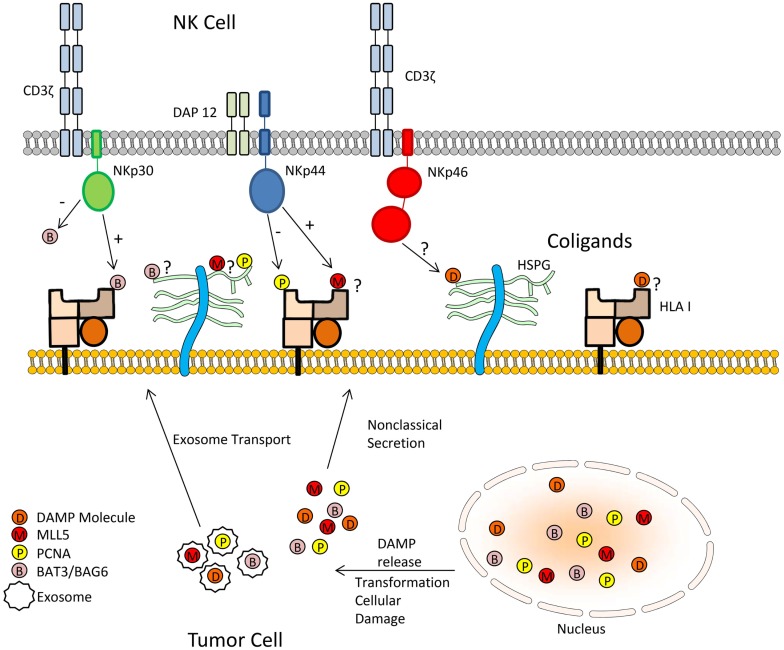
**Mechanism by which tumor cells may inhibit natural cytotoxicity through NCR recognition by DAMPs**. The association of DAMPs with HSPGs, HLA I, or other potential co-ligands may form larger complex ligands for the members of the NCR family.

We postulate that DAMPs may serve as the missing link in NCR-mediated recognition of tumor cells. The association of DAMPs with HSPGs, HLA I, or other potential co-ligands may form larger complex ligands for the members of the NCR family (Figure 1). Human leukocyte antigen-B-associated transcript 3 (Bat3), also known as BAG-6, could be considered a DAMP due to its release from tumor cells (62). Bat3 is typically located in the nucleus where it plays an essential role in controlling the acetylation of p53 in response to cellular DNA damage (62). However, upon non-lethal heat shock, nuclear Bat3 relocates to the cell membrane of tumors where it serves as a ligand for NKp30 (62, 63). Interestingly, this study found Bat3 colocalizes with HLA I, suggesting opposition to previous reports that NCRs do not associate with HLA I molecules (29, 63, 70).

Like Bat3, PCNA and MLL5 are located in the nucleus and cytoplasm. Additionally, all three molecules are intricately involved in processes regulating the cell cycle and/or DNA repair mechanisms. Thus, their presence on the cell surface may indicate intracellular stress related to DNA damage or improper cell cycle control, qualifying these molecules as DAMPs. This suggests that the NCRs may be pattern recognition receptors, which recognize DAMPs sequestered to the cell surface. Taken into context with other studies, the NCRs potentially have the ability to interact with HLA I, HSPGs, or other cell surface molecules as co-ligands in conjunction with soluble proteins, such as Bat3, PCNA, MLL5, or other DAMPs on the cell surface of tumors (62). Therefore, the NCRs may recognize DAMPs on the cell surface in association with a docking protein. Furthermore, the NCRs may be directly recognizing the C-terminal ends of DAMPs. NKp30 was recently shown to recognize the C terminus of Bat3 (71). In a similar manner, NKp44 also recognizes the C terminus of the MLL5 ligand (53). The molecular details of interaction between NKp44 and PCNA have yet to be determined, but the above precedents suggest that interaction may occur at the C terminus of PCNA.

Natural cytotoxicity receptors ligands consisting of a DAMP and a co-ligand may add complexity in understanding how the NCRs regulate NK cell effector function. The NCRs were originally believed to be strictly activating NK cell receptors. However, NKp44 and NKp30 have recently been shown to exhibit both inhibitory and activating functions. NKp44 recognizes cell surface PCNA in an inhibitory manner while a soluble C-terminal fragment of Bat3 inhibits NK cell function via NKp30 (15, 71). However, recognition of MLL5 by NKp44- and NKp30-mediated recognition of Bat3 sequestered to the cell surface activates NK cell effector functions (53, 62, 63). Thus, modulation of NK cell activity via the NCRs could depend on the DAMP molecule, the co-ligand sequestering the DAMP, or the lack of a coligand and the soluble nature of the DAMP. NKp44 presents a more special case since it contains a functional ITIM-like sequence in its cytoplasmic tail. Due to the dual nature of NKp44 signaling, it will be of interest to determine if recognition of the DAMP, either PCNA or MLL5, the coligand, potentially an HSPG or HLA I, or the motif as a whole is responsible for inhibition or activation of NK cytotoxicity. Neither NKp30 nor NKp46 has been reported to contain an ITIM sequence. However, an immunosuppressive isoform of NKp30 resulting from a single-nucleotide polymorphism in the 3′-untranslatable region has been reported (72). Whether the divergence of NCR function depends on the individual DAMP molecule recognized or the binding of DAMP molecules to a specific coligand remains to be elucidated.

## Concluding Remarks

Recent studies reveal a novel function for DAMP molecules, or proteins, which are located and function intracellular, but somehow localize to the extracellular membrane despite lacking a traditional secretory leader sequence. These proteins are released by cells, which have become injured in the absence of infection due to ischemia, hypoxia, transformation, chemotherapy, DNA damage, or other trauma. Analogous to TLRs recognizing pathogen-associated molecular patterns, the NCRs may represent a class of NK cell receptors that participate in pattern recognition of DAMP molecules, whose identities may reflect the intracellular health of a cell, particularly in regards to DNA damage or instability, in addition to the traditional method of HLA I presenting self peptide. In this manner, HLA I, HSPGs, or other co-ligands may present DAMP molecules for identification by the NCRs, which would then regulate NK cell function. Like other NK cell receptors, the NCRs undoubtedly recognize multiple ligands, which may be cell surface transmembrane proteins, like the recognition of B7-H6 molecule by NKp30 (73). Knowledge of the identities of NCR ligands and nature of DAMP molecules that bind to HLA I, HSPGs, or other cell surface molecules to form complex ligands for the NCRs will shed light on NK cell recognition of target cells under healthy and disease conditions and offer novel therapeutic targets.

## Conflict of Interest Statement

The authors declare that the research was conducted in the absence of any commercial or financial relationships that could be construed as a potential conflict of interest.
